# Establishment of a Comprehensive Quality Evaluation Model for Japonica Rice With Different Degrees of Milling

**DOI:** 10.1002/fsn3.70908

**Published:** 2025-09-04

**Authors:** Shan Zhang, Bin Hong, Di Yuan, Shan Shan, Jingyi Zhang, Shiwei Gao, Qing Liu, Dapeng Chen, Weiwei Yin, Chuanying Ren

**Affiliations:** ^1^ Food Processing Research Institute Heilongjiang Academy of Agricultural Sciences Harbin China; ^2^ Heilongjiang Province Key Laboratory of Food Processing Harbin China; ^3^ Heilongjiang Province Engineering Research Center of Whole Grain Nutritious Food Harbin China; ^4^ Suihua Branch of Heilongjiang Academy Agricultural Sciences Suihua China; ^5^ Heilongjiang Vocational College of Agricultural Technology Jiamusi China

**Keywords:** comprehensive analysis, degree of milling, *japonica* rice, quality evaluation

## Abstract

Japonica is considered one of the better tasting varieties, so it is important to balance the quality and taste of japonica rice produced by moderate processing. This study analyzed the changes in bioactive components, heavy metal elements, and sensory quality of northern japonica rice after gradient milling, and constructed a comprehensive quality evaluation model for japonica rice with different degrees of milling. The results showed that as the degree of milling (DOM) increased from 0% to 10%, the bioactive components in japonica rice decreased, with dietary fiber (3.67%–0.71%), total phenols (0.73–0.24 mg/g), and vitamin B_2_ (0.063–0.025 mg/kg) being markedly reduced. Moreover, heavy metal elements such as inorganic arsenic, cadmium (Cd), and mercury also showed a downward trend. In sensory analysis, the taste quality of japonica rice with different degrees of milling showed an upward trend. Correlation analysis was further used to discuss the comprehensive indicators of gradient milled japonica rice, and the results showed that there were varying degrees of correlation among the indicators. Therefore, principal component analysis (PCA) was used to remove the overlapping information, and three principal components were extracted with a cumulative contribution rate of 91.67%. This corresponds to the results of cluster analysis, which divided the rice into three categories based on indicators and varieties. Finally, we obtained the edible quality evaluation model of japonica rice with different degrees of milling as *Z* = 0.81*F*_1_ + 0.13*F*_2_ + 0.07*F*_3_. Simultaneously, we determined that the comprehensive quality of japonica rice with a milling accuracy of 9% (J‐9) was the highest in gradient milling, which provided a theoretical basis for moderate processing of japonica rice.

## Graphical abstract

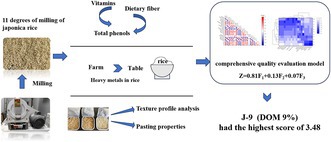

## 1 Introduction

Rice ( *Oryza sativa* L.) plays a pivotal role in human development, with more than half of the world's farmers cultivating rice and more than 3.5 billion people consuming rice as a staple food (Mawia et al. 2021). Specifically, China is the largest producer and consumer of rice worldwide, where 60% of the population consumes rice as a primary nutritional source. In terms of grain shape and quality, rice is divided into two categories: indica and japonica. Japonica rice is mainly grown in northern China, the middle and lower reaches of the Yangtze River, and in the lower‐temperature, high‐altitude areas of the Yunnan‐Guizhou Plateau. Within these regions, rice is full and rounded, crystalline, translucent, and has a better flavor quality.

Compared to white rice, in addition to basic nutrients such as protein, fat, and starch, brown rice contains many unique bioactive components, including dietary fiber, vitamins, and total phenols. These active ingredients have certain health benefits for human blood sugar and lipid levels. However, most of these components are transferred to by‐products during the processing into white rice (Liu et al. 2021; Ma, Yi, et al. 2020). Brown rice undergoes moderate processing to reduce its nutritional loss and energy consumption, a practice that has been actively promoted in the rice industry in recent years (Li et al. 2025). Therefore, the degree of rice milling is well known as an indicator to measure the amount of bran removed during the rice milling process. In 2018, the national standard GB/T1354 refined the degree of rice milling into two categories: fine milling and suitable milling, while the USDA Federal Grain Inspection Service divided it into four more detailed qualitative levels: well‐milled, reasonably well‐milled, lightly milled, and undermilled. Although moderate processing is feasible, differences in appearance and taste between moderately processed rice and white rice persist, and the retention of contamination by moderate processing can lead to rancidity during storage (Miho et al. 2018). Therefore, it is crucial to ensure a balance between the taste and quality in moderately processed rice.

The evaluation of food quality through taste and sensory assessment is common practice; however, this method involves a degree of subjectivity and uncertainty. With the help of statistical data software, principal component analysis (PCA), correlation analysis (CRA), cluster analysis (CA), screening of quality indicators, and establishment of comprehensive quality evaluation models are simplified. Shi et al. (2021) used PCA to evaluate the comprehensive quality of seven rice varieties under 12 nitrogen fertilizer treatments with changes in 17 qualities. It was found that rice quality was optimal under low‐nitrogen conditions. Another study used PCA to examine the relationship between variations in the biochemical composition of the endosperm and the nutritional and edible qualities of rice, thereby providing insights to guide rice breeding and cultivation (Ma, Wang, et al. 2020). Furthermore, PCA is commonly used for evaluating other foods and fruits. PCA and CRA determined that the sensory qualities of pork cooked using different methods (boiling, scalding, and roasting) were different. Key quality indicators of pork were identified, and comprehensive quality evaluation equations were developed for pork under the corresponding cooking conditions (Duan et al. 2023). Nie et al. (2019) determined the optimal harvesting period for Majiayou pomelo using PCA, which provided a theoretical basis and practical foundation for fruit harvesting and the maintenance of functional components.

To date, there is a lack of relevant data to support the comprehensive evaluation of the eating quality of japonica rice with different DOM. Additionally, balancing the quality and taste of japonica rice produced by moderate processing remains an issue that requires further investigation. Therefore, 11 types of japonica rice with varying degrees of DOM were investigated, and 20 key evaluation indices were selected, including hardness, adhesiveness, springiness, cohesiveness, gumminess, chewiness, resilience, pasting temperature, peak viscosity, trough viscosity, final viscosity, breakdown, setback, inorganic arsenic, Cd, total phenols, dietary fiber, Vitamin B_2_ (VB_2_), Vitamin B_1_ (VB_1_) and Vitamin E (VE). Additionally, PCA with CRA and CA was performed for a comprehensive evaluation. Therefore, this study aimed to provide a theoretical foundation and data support for the moderate processing and industrial application of japonica rice.

## 2 Materials and Methods

### 2.1 Materials

Suijing309 (length: 4.43 ± 021 mm, length to breadth ratio: 1.68 ± 0.09, thousand grain weight: 21.27 ± 0.38 g), provided by Suihua Branch of Heilongjiang Academy Agricultural Sciences and harvested in 2022, was used as the experimental material for this study. All chemicals used were of analytical grade.

### 2.2 Milling Process

A huller was used to obtain brown rice that was subsequently ground with a grain polisher (TM05, Satake Machinery [Suzhou] Co. Ltd.) to obtain 11 samples with DOM of 0%, 1% ± 0.1%, 2% ± 0.1%, 3% ± 0.1%, 4% ± 0.2%, 5% ± 0.2%, 6% ± 0.3%, 7% ± 0.3%, 8% ± 0.2%, 9% ± 0.1%, and 10% ± 0.1%, within time controls. The milling process adopted the method previously reported by Shen et al. (2023). These samples are named J‐0, J‐1, J‐2, J‐3, J‐4, J‐5, J‐6, J‐7, J‐8, J‐9, and J‐10. The milled samples were sieved with a 10‐mesh screen to remove rice bran, packed in sealed polyethylene bags, and stored in a refrigerator at 4°C until further analysis.

$$\mathrm{DOM}\;\left(\%\right)=100—\left[\;\text{weight of milled rice}/\text{weight of brown rice}\right]$$

### 2.3 Dyeing

Rice samples (12 g) with different DOM were placed in a 90 mm diameter petri dish. After cleaning, eosin and methylene blue solutions were added for 2 min, the dyeing solvent was removed, and the samples were washed three times with 80% ethanol. The samples were dried and placed on the scanning plate of a rice appearance quality detector to capture the scanned images (Zhao et al. 2023).

### 2.4 Morphology Observation

Cut the freeze‐dried rice and apply gold sputtering coating to the obtained sections and surfaces. The microstructure was observed under a scanning electron microscope (SEM) (TESCAN MIRA LMS, Czech Republic, Europe) with magnifications of 750× (Ye et al. 2024).

### 2.5 Nutritional Components Analysis

#### 2.5.1 Extraction and Determination of Total Phenols

The total phenol content was measured according to the method described by Zhang et al. (2024), with slight modifications. Briefly, 1 g of rice powder was mixed with 20 mL of ethanol and subjected to ultrasonic treatment at 60°C for 30 min. The mixture was then centrifuged at 12,000 rpm for 10 min in a 2‐16P centrifuge (SIGMA, Germany) to separate the supernatant. A 2.5 mL aliquot of the supernatant was reacted with 2.5 mL of Folin reagent and 2.5 mL water for 2 min. Subsequently, 10 mL of Na_2_CO_3_ was added, and the mixture was allowed to react for 1 h at room temperature. After the reaction, absorbance was measured at 760 nm using a spectrophotometer (Varian, USA).

#### 2.5.2 Determination of Vitamins

The vitamin B_1_, vitamin B_2_, and vitamin E content of the rice flour were measured according to the AOAC methods (AOAC 2015). Measure the absorbance of the vitamin B_1_, vitamin B_2_, and vitamin E extract solution using a spectrophotometer at 360, 510, and 520 nm, respectively.

#### 2.5.3 Determination of Dietary Fiber Content

Dietary fiber content was determined using an enzymatic gravimetric method. The defatted samples were treated successively with alpha‐amylase (heat stable), protease, and amyloglucosidase. They were then washed and dried with ethanol to obtain a residue. The total dietary fiber content of rice with different DOM was obtained by converting it into ash and protein (Wang et al. 2021).

### 2.6 Safety Quality Analysis

An atomic absorption spectrophotometer was used to measure the concentrations of four heavy metal elements, inorganic arsenic, lead, Cd, and mercury, in different DOM rice samples (Perkin Elmer, USA). Initially, 0.2 g (accurate to 0.001 g) of the solid sample was digested using a microwave digestion system (PreeKem, China) with 5 mL of HNO_3_. After digestion, the acid solution was rushed to approximately 1 mL at 140°C. Deionized water was added to the digested samples to a final volume of 25 mL. A control sample was evaluated simultaneously (Hasan et al. 2022).

### 2.7 Cooked Quality Analysis

#### 2.7.1 Pasting Properties

The pasting properties of rice were determined using a viscometer with a thermostat water bath (Brookfield, DV2TLV, USA). The rice flour suspension at the concentration of 10% (w/w) was prepared and mixed evenly. A programmed heating and cooling cycle was conducted, during which the sample was held at 50°C for 1 min, then heated to 95°C and maintained at 95°C for 5 min before cooling to 50°C. Pasting temperature, peak viscosity, trough viscosity, final viscosity, breakdown, and setback were recorded (Xu et al. 2021).

#### 2.7.2 Textural Profile Analysis

Textural profile analysis (TPA) of the cooked rice was performed using a texture analyzer (TA.XT.plus, UK). Texture was determined according to the method described by Ni et al. (2019). A P/36R cylindrical probe was used to compress three rice grains. The specifications were as follows: Pre‐test speed 1.0 mm/s, Test speed 5.0 mm/s, Post‐test speed 5.0 mm/s, Target mode Strain, Strain 45%, Time 5.0 s, Trigger force 5.0 g. Hardness, adhesiveness, springiness, cohesiveness, gumminess, chewiness, and resilience were the evaluated indices to determine rice texture.

#### 2.7.3 Taste Measurement

Eight grams of the prepared rice were placed in a stainless‐steel ring and pressed into a rice cake using a mold. The rice cakes were placed in a flavor analyzer (SATAKE, Japan) for measurement. Each sample was measured thrice, and each sample was measured once on the front and back faces to obtain three indicators: appearance, taste, and overall score (Ma, Wang, et al. 2020).

### 2.8 Statistical Analysis

SPSS (version 23.0; IBM, USA) and Origin 2021 (Origin Lab, USA) were used to analyze the data. CRA, CA, and data were analyzed and plotted using Origin 2021. SPSS was used to analyze significant differences (SDA) and principal components (PCA). Samples were analyzed in triplicate, and the results are expressed as the mean ± standard deviation.

## 3 Results and Discussion

### 3.1 Milling Quality of Rice Grains

**FIGURE 1:**
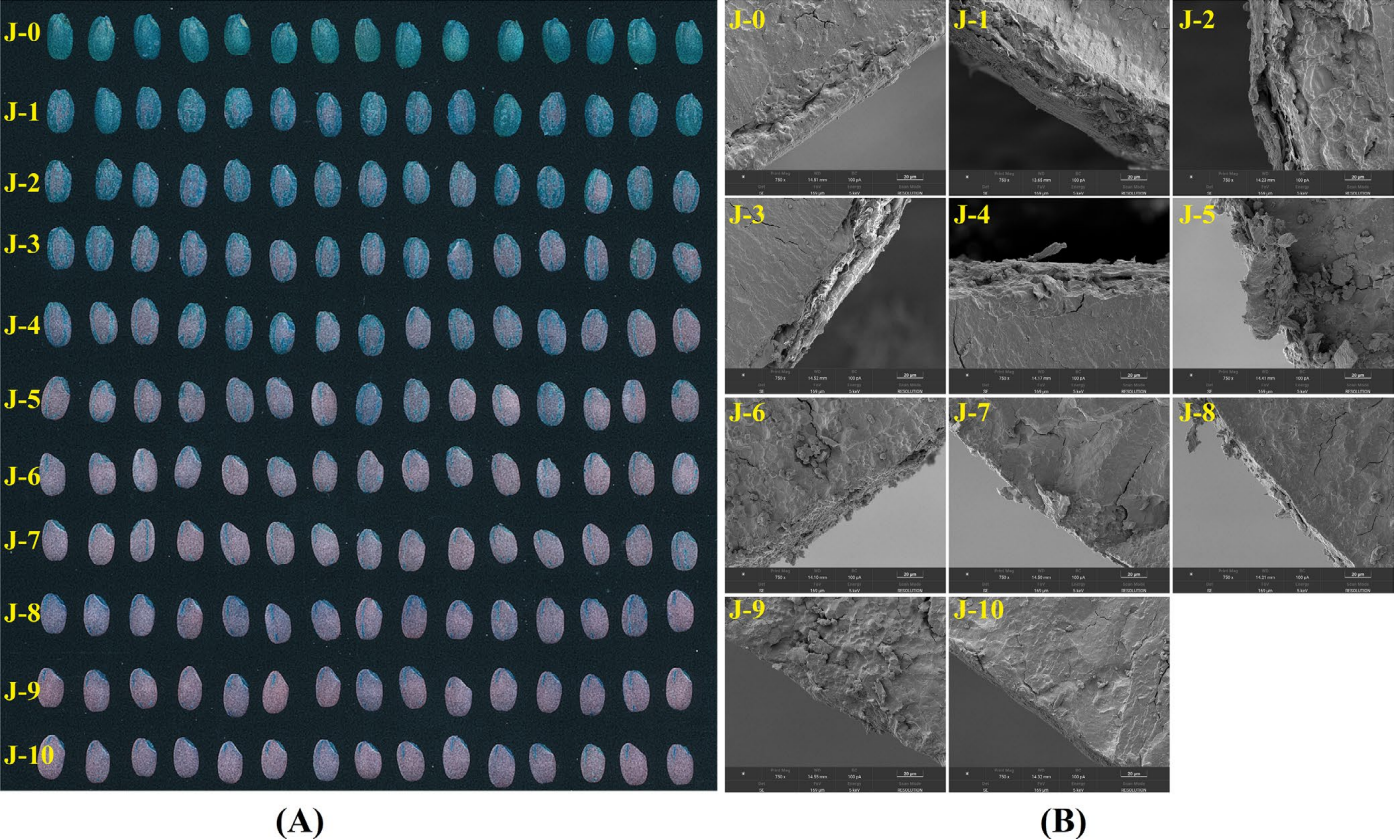
Staining images and transverse sections of rice grains in different DOM, J‐0 to J‐10 are rice grain samples at DOM of 0%, 1%, 2%, 3%, 4%, 5%, 6%, 7%, 8%, 9%, and 10%, respectively.

The dyeing method leverages the different affinities of rice bran, endosperm, and germ to eosin Y and methylene blue. After dyeing, the bran and embryo appear bluish green, while the endosperm takes on a purplish‐red color (Serna‐Saldívar 2012). As shown in Figure 1A, the rice grains gradually transitioned from green to pink as the milling degree increased. When the DOM was from 0% to 4%, the rice grains appeared blue‐green. As processing precision increased, the green color gradually faded, indicating a reduction in the bran layer. When the DOM was from 9% to 10%, a small amount of bran was present near the embryo, appearing blue‐green. The portions were pink, indicating that the rice bran had been completely milled, and the endosperm was fully exposed.

The surface and transverse sections of gradient milling of rice were observed through a SEM (Figure 1B J‐0–J‐10, Figure S1 (a)–(k)). It can be seen that the surface of brown rice (J‐0) is smooth and glossy, presenting a frosted glass‐like appearance, and its cross‐section has a thick layer of rice bran. With the DOM increased, the surface of rice gradually becomes rice bran due to the pressure, and the outer layer becomes uneven, filled with broken sheet‐like seed coat residues and scattered granular paste layers, and some rice grains have cracks.

### 3.2 Bioactive Components of Cooked Rice With Different DOM

**FIGURE 2:**
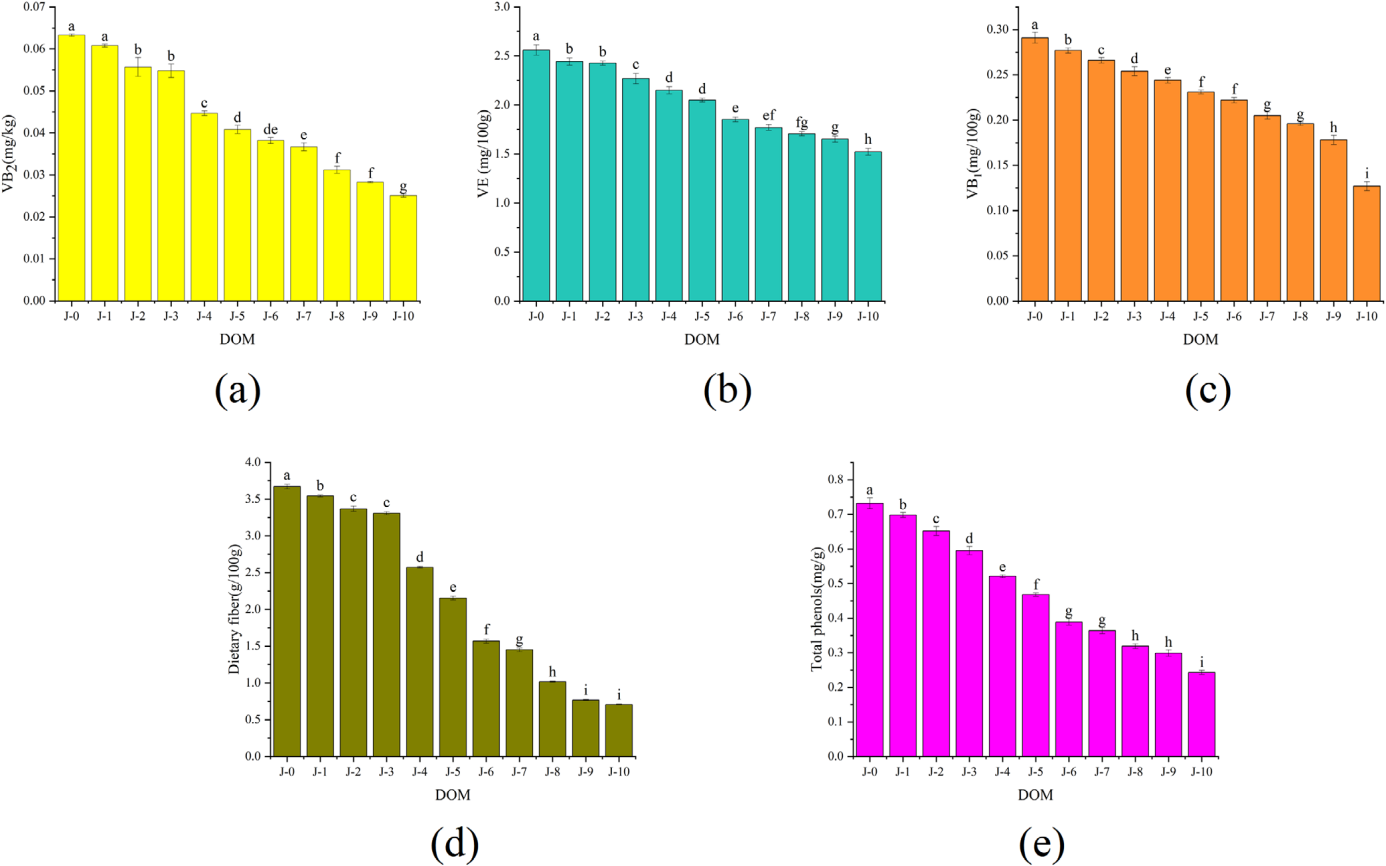
Effect of different DOM on bioactive components VB_2_ (a), VE (b), VB_1_ (c), dietary fiber (d), total phenols (e), contents of rice. Different letters indicate significant differences (*p* < 0.05).

The white rice obtained after milling brown rice can result in the loss of many bioactive components according to some literature reports. Therefore, an analysis was conducted on some unique nutritional components in the bran, mainly vitamins, total phenols, and dietary fiber. Vitamin B_2_, also known as riboflavin, the average requirements and population reference intakes (PRIs) for all groups were 1.3 and 1.6 mg/day in the standards issued by the European Union, respectively (EFAS, 2017; https://www.efsa.europa.eu/en/efsajournal/pub/4919). Therefore, brown rice is a good source of VB_2_ (Liu et al. 2017). However, milling gradually reduced the VB_2_ content in brown rice (Figure 2c). VB_2_ in brown rice (J‐0) was 0.063 mg/kg, and the level of VB_2_ decreased to 0.025 mg/kg in J‐10 rice. Therefore, the removal of rice bran caused a significant loss of VB_2_. In addition, vitamin B_1_ and vitamin E were significantly reduced (*p* < 0.05) in the grinding accuracy of 10% (J‐10) (Figure 2b,c), which is consistent with previous research results (Chaijan and Panpipat 2020).

Dietary fiber, which comprises polysaccharides, oligosaccharides, lignin, and associated plant substances, is a good nutritional package (Elleuch et al. 2011). The dietary fiber content in rice grains decreased from 3.67% (J‐0) to 0.71% (J‐10) (Figure 2d), confirming a higher amount of fiber in the outer layers of rice (Kalpanadevi et al. 2018). Total phenols are present in three forms within the germ and bran layers of rice (Ravichanthiran et al. 2018). The results indicated a consistent decrease in the total phenol content by approximately 50% in J‐10 grains compared to that in brown rice (J‐0) (Figure 2e). Furthermore, milling results in a consistent decrease in bioactive components, as total phenols are associated with several physiological functions and health benefits.

### 3.3 Safety Quality of Cooked Rice With Different DOM

Currently, heavy metal contamination is a major quality and safety concern for grains, with little improvement. Lead, cadmium, mercury, and arsenic are four common heavy metal elements in rice, all of which can cause varying degrees of damage to the human body. In particular, inorganic in arsenic is a carcinogenic element that can cause respiratory inflammation, kidney dysfunction, lung dysfunction, bone disease, cancer, and high blood pressure (Ge et al. 2020). In studies on the distribution pattern of heavy metal pollution in rice grains, it was found that the heavy metal content in bran is significantly higher than that in rice husks and endosperms (Chen et al. 2022). It can be seen from Table 1 that as the milling degree increases, the four heavy metal elements in rice gradually decrease. Among them, lead was not detected in Suijing 309, and mercury was also not detected when the milling degree was greater than 2%. This may be related to the soil in the planting area. Inorganic arsenic and cadmium continued to decrease with the increase of DOM, decreasing from 0.174 mg/kg (J‐0) to 0.087 mg/kg (J‐10) and from 0.00577 mg/kg (J‐0) to 0.00372 mg/kg (J‐10), respectively. Therefore, milling is an important physical method for reducing heavy metals.

**TABLE 1:** Heavy metals of rice with different DOM.

DOM	Lead (mg/kg)	Cd (mg/kg)	Inorganic arsenic (mg/kg)	Mercury (mg/kg)
J‐0	—	0.00577 ± 0.00006a	0.174 ± 0.004a	0.00169 ± 0.00004a
J‐1	—	0.00556 ± 0.00005b	0.173 ± 0.003ab	0.00158 ± 0.00005b
J‐2	—	0.00534 ± 0.00005c	0.164 ± 0.004b	0.00124 ± 0.00003c
J‐3	—	0.00483 ± 0.00009d	0.151 ± 0.003c	—
J‐4	—	0.00465 ± 0.00003e	0.143 ± 0.003cd	—
J‐5	—	0.00447 ± 0.00003f	0.135 ± 0.004de	—
J‐6	—	0.00412 ± 0.00005g	0.131 ± 0.003ef	—
J‐7	—	0.00404 ± 0.00002gh	0.122 ± 0.003f	—
J‐8	—	0.00392 ± 0.00004hi	0.096 ± 0.002g	—
J‐9	—	0.00383 ± 0.00002ij	0.091 ± 0.002g	—
J‐10	—	0.00372 ± 0.00005j	0.087 ± 0.005g	—

*Note:* Different letters (a‐j) in the same column indicate significant difference (*p* < 0.05).

### 3.4 Eating Quality of Cooked Rice With Different DOM

#### 3.4.1 Pasting Properties

The pasting properties of the rice subjected to milling are listed in Table 2. With an increase in milling, the peak viscosity, trough viscosity, final viscosity, breakdown, and setback increased significantly (*p* < 0.05). Conversely, the pasting temperature was reduced from 66.0°C to 62.8°C (*p* < 0.05). Peak viscosity is defined as the highest viscosity that can be achieved by rapid shearing of rice flour at high temperatures, reflecting the ability of starch granules to swell and bind water during the pasting process (Ukpong et al. 2021). Rice bran decreased proportionately when the DOM increased, and the mechanical damage of starch granules from the milling process caused the absence of network structures. Due to the increased number of free hydroxyl groups present, permitting the peak viscosity to gradually increase from 3591.0 to 7802.3 cp. Studies have shown that consumers are more likely to accept rice with a higher peak viscosity (Charoenthaikij et al. 2021). Breakdown analysis demonstrated the collapse of starch molecules during the cooking process, and Table 1 indicates that J‐10 (4920.0 cp) exhibited a higher resistance to shear stress and heat. It has been reported that a higher breakdown is one of the characteristics of rice with better eating quality (Ukpong et al. 2023). The retrogradation ability of starch upon cooling is indicated by its setback viscosity (Ukpong et al. 2024). The arrangement of rice starch molecules at low temperatures is easier, and the gel strength is enhanced without the protection of rice bran. Thus, J‐10 (4829.0 cp) exhibited a stronger gelling capacity than that of J‐0 (2828.0 cp). It is worth noting that when the milling degree of rice is above 8%, there is a fluctuation in the gelatinization temperature of rice. This may be because the gelatinization of rice is closely related to the presence and concentration of substances such as protein, fat, total phenols, and dietary fiber (Zhu et al. 2016). These substances compete for water or partially bind with starch, thereby increasing the gelatinization temperature of rice (Mandala and Bayas 2004). In this experiment, the concentration of these substances increased with the degree of milling, which affected the gelatinization temperature. But overall, with an increase in DOM, rice bran was gradually reduced and the endosperm was exposed, which led to an improvement in the pasting properties and taste of rice. This corresponds with the taste value results.

**TABLE 2:** Pasting properties and texture properties of rice with different DOM.

DOM	Pasting temperature (°C)	Peak viscosity (cp)	Trough viscosity (cp)	Final viscosity (cp)	Breakdown (cp)	Setback (cp)	Hardness (g)	Adhesiveness (g.s)	Springiness	Cohesiveness	Gumminess (g)	Chewiness (g)	Resilience
J‐0	66.0 ± 0.1a	3591.0 ± 72.3a	1730.0 ± 7.9 g	4558.0 ± 25.7j	1861.0 ± 65.6i	2828.0 ± 17.8f	674.24 ± 53.24a	−0.96 ± 0.56a	0.93 ± 0.07a	0.65 ± 0.02a	435.25 ± 37.70a	405.89 ± 63.28a	0.60 ± 0.07a
J‐1	63.2 ± 0.2c	4341.3 ± 88.4b	1843.3 ± 20.6f	5206.7 ± 75.4i	2498.0 ± 71.0 h	3363.3 ± 95.3e	547.51 ± 21.48b	−1.95 ± 0.11ab	0.92 ± 0.02a	0.61 ± 0.03ab	353.52 ± 17.67b	307.43 ± 19.96b	0.57 ± 0.02a
J‐2	62.1 ± 0.1d	5676.3 ± 45.5d	2315.0 ± 70.8d	6861.7 ± 22.5f	3361.3 ± 107.6f	4546.7 ± 93.1b	505.10 ± 7.27c	−2.69 ± 0.19bc	0.89 ± 0.01a	0.62 ± 0.03ab	311.35 ± 17.08c	277.09 ± 12.72b	0.59 ± 0.01a
J‐3	62.1 ± 0.3d	4936.7 ± 14.0c	2074.7 ± 6.7e	6239.0 ± 8.9 h	2862.0 ± 20.1 g	4164.3 ± 12.2d	480.27 ± 12.49c	−3.26 ± 0.05 cd	0.90 ± 0.02a	0.59 ± 0.03ab	283.32 ± 18.65c	254.18 ± 11.92bc	0.54 ± 0.02a
J‐4	61.8 ± 0.1de	5878.0 ± 10.8e	2327.0 ± 36.3d	6682.7 ± 49.7 g	3551.0 ± 37.2e	4355.7 ± 44.5c	360.02 ± 15.57de	−3.92 ± 0.61de	0.83 ± 0.14a	0.58 ± 0.02b	209.23 ± 13.6de	173.84 ± 35.96def	0.55 ± 0.03a
J‐5	61.7 ± 0.1de	5848.0 ± 20.0e	2297.7 ± 26.6d	7047.3 ± 28.2e	3550.3 ± 35.6e	4749.7 ± 51.6a	336.36 ± 6.35ef	−5.02 ± 0.28ef	0.94 ± 0.04a	0.60 ± 0.01ab	203.02 ± 7.29de	191.23 ± 14.29de	0.56 ± 0.06a
J‐6	61.5 ± 0.1e	6066.3 ± 36.0f	2328.3 ± 17.9d	7101.3 ± 42.5de	3738.0 ± 52.0d	4773.0 ± 28.2a	311.16 ± 6.39 fg	−6.01 ± 0.40f	0.91 ± 0.01a	0.59 ± 0.01ab	184.25 ± 2.64ef	168.24 ± 1.38def	0.58 ± 0.04a
J‐7	61.0 ± 0.2f	6727.0 ± 28.1 g	2451.3 ± 48.4c	7195.0 ± 22.7d	4275.7 ± 23.5c	4743.7 ± 58.3a	293.97 ± 8.63gh	−7.91 ± 0.47 g	0.90 ± 0.01a	0.59 ± 0.01ab	172.07 ± 5.39efg	155.47 ± 2.94ef	0.53 ± 0.03a
J‐8	64.1 ± 0.2b	7061.7 ± 83.1 h	2677.3 ± 26.5b	7386.3 ± 25.4c	4384.3 ± 80.9c	4709.0 ± 5.6a	260.59 ± 7.12hi	−9.02 ± 0.21 g	0.92 ± 0.03a	0.59 ± 0.04ab	154.75 ± 13.21 fg	141.54 ± 7.91ef	0.56 ± 0.04a
J‐9	64.4 ± 0.2b	7478.3 ± 40.3i	2836.7 ± 15.5a	7551.0 ± 37.7b	4641.7 ± 53.1b	4714.3 ± 36.5a	249.98 ± 4.43i	−10.60 ± 0.63 h	0.93 ± 0.01a	0.58 ± 0.03ab	144.35 ± 9.40 g	133.91 ± 7.97f	0.52 ± 0.02a
J‐10	62.8 ± 0.2c	7802.3 ± 56.5j	2882.3 ± 47.1a	7711.3 ± 17.2a	4920.0 ± 46.7a	4829.0 ± 46.8a	378.98 ± 16.47d	−4.08 ± 0.63de	0.91 ± 0.03a	0.62 ± 0.02b	234.02 ± 16.58d	213.61 ± 18.36 cd	0.58 ± 0.05a

*Note:* Different letters in the same column indicate significant difference (*p* < 0.05).

#### 3.4.2 Texture Profile Analysis

The textural characteristics of the rice with different DOM are presented in Table 2. With an increase in DOM, the hardness, gumminess, and chewiness of rice decreased, whereas adhesiveness increased. With an increase in DOM, the hardness decreased gradually in the range of 674.24–249.98 g. This decrease was due to peeling, causing damage to the bran layer of rice, resulting in an increase in the speed of water diffusion and the degree of starch gelatinization and swelling (Zhang et al. 2023). Adhesiveness significantly contributes to the textural properties of rice. Adhesiveness refers to the degree of adhesion of a sample to a probe when it is pulled away from the sample surface (Cilurzo et al. 2012). In this experiment, the adhesiveness of J‐0 was −0.96 g.s and J‐9 was −10.60 g.s, showing an overall upward trend. However, similar to the hardness results, the adhesiveness of J‐10 rice decreased, which may be related to the leaching of starch solids. Research has shown that the amylose in solids is positively correlated with the adhesiveness of rice (Li et al. 2021). With the increase of DOM, the solids in rice are completely transferred to the rice soup after leaching, as the decrease in surface attached solids leads to changes in adhesiveness. At the same time, the changes in rice texture characteristics are also related to the complex changes in starch‐lipid complexes (Shen et al. 2024). Anyway, the adhesiveness of milled rice is enhanced, which is closely related to the increased starch leaching caused by damage and rupture of the rice surface during the cooking process. Hardness and adhesiveness are key indicators of rice, and a decrease in hardness and increase in adhesiveness indicate an improvement in the overall taste of rice.

#### 3.4.3 Sensory Measurement of Cooked Rice With Different DOM

**FIGURE 3:**
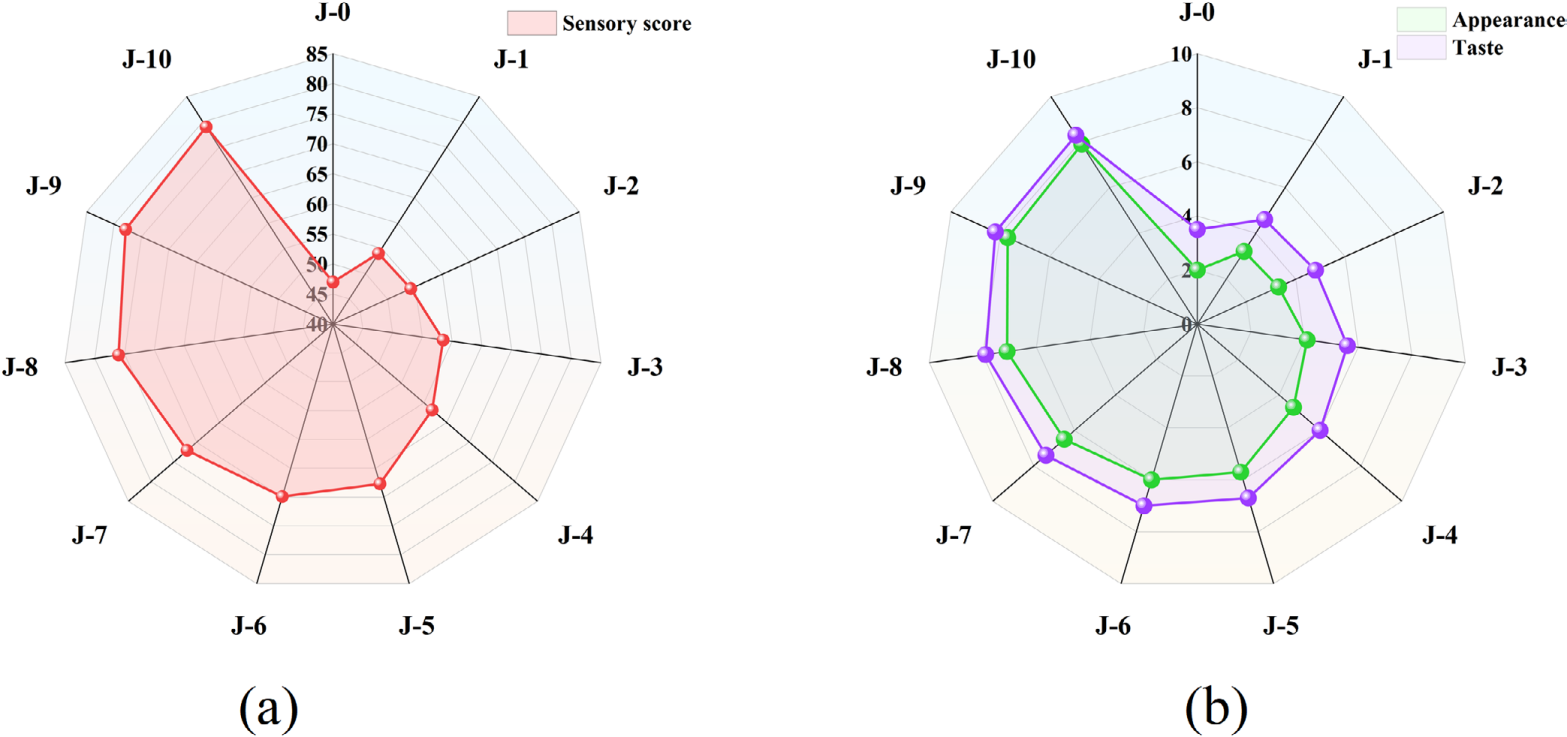
Sensory evaluation (a) and overall score (b) of cooked rice with different DOM.

The results of sensory measurements are shown in Figure 3. The taste score of rice shows an upward trend with the increase of DOM, which mainly manifested as a significant improvement in appearance, taste, and sensory score. As shown in Figure 3b, the appearance score of J‐0 (brown rice) was 2.0, while the J‐10 (DOM 10%) reached 7.9, indicating that the milling process can destroy the bran layer and made the endosperm exposed to an increasing in DOM, thus improving whiteness and providing a significant improvement in overall appearance quality (Zhao et al. 2023). In addition, due to the fact that rice bran contains 35%–50% dietary fiber, which will affect the taste of rice (Liu et al. 2023). Thus, when DOM reached 10%, the rice exhibited favorable taste quality, with a sensory score of 79, which was nearly double that of brown rice (Figure 3a).

### 3.5 Comprehensive Quality Analysis and Evaluation of Japonica Rice

#### 3.5.1 Correlation Analysis

**FIGURE 4:**
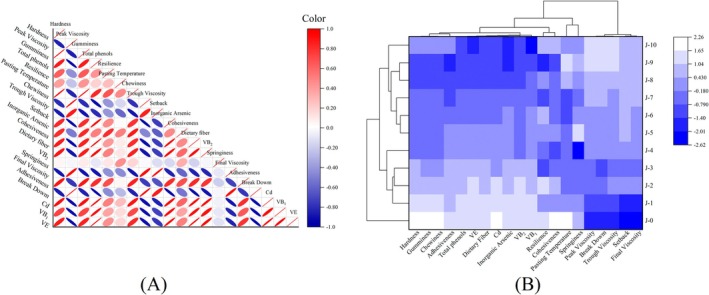
Correlation (A) and cluster analysis (B) of rice with different DOM.

There may be positive or negative correlations between different nutrients. Pearson correlation analysis can reveal the degree of correlation between two parameters (Song et al. 2023). In this study, the correlation analysis of the obtained data revealed significant or extremely significant (*p* < 0.05, *p* < 0.01) correlations between the different qualities of rice and DOM (Figure 4A). Hardness was significantly negatively correlated with peak viscosity, trough viscosity, setback, final viscosity, and breakdown (*r* = −0.86, −0.82, −0.87, −0.89, −0.87; *p* < 0.01), and significantly positively correlated with VB_2_, total phenols, and dietary fiber (*r* = 0.89, 0.88, 0.87; *p* < 0.01). The total phenol content was significantly and positively correlated with dietary fiber content (*r* = 0.99; *p* < 0.01). The correlation between the evaluation indicators of rice with different DOM showed a significant or extremely significant relationship, resulting in overlapping information, and each indicator had its own advantages and disadvantages. Therefore, it is necessary to use principal component analysis to reduce the overlap of the quality indicators of rice and improve the accuracy of the evaluation.

#### 3.5.2 Principal Component Analysis

The results of the PCA are presented in Table 3, each of which had a characteristic root > 1. The total contribution of the three principal components exceeded 85%, which could reflect most of the information on rice with different DOM qualities (Ni et al. 2019). Therefore, selecting the first three principal components was most appropriate, with a cumulative variance contribution rate of 91.67% (Nie et al. 2019). PC1 had the greatest impact on rice quality, explaining 73.93% of the variation in the rice quality indicators, whereas PC2 and PC3 explained 11.49% and 6.25% of the variance, respectively.

**TABLE 3:** Principal component analysis of quality indicators of rice.

Standardized	Indexes	Principal components		
		1	2	3
X_1_	Hardness	0.958	0.167	−0.118
X_2_	Adhesiveness	0.861	−0.047	−0.414
X_3_	Springiness	−0.025	−0.678	−0.41
X_4_	Cohesiveness	−0.692	−0.616	0.318
X_5_	Gumminess	0.955	0.218	−0.108
X_6_	Chewiness	0.943	0.280	−0.088
X_7_	Resilience	−0.586	−0.414	0.561
X_8_	Pasting Temperature	0.318	0.734	0.415
X_9_	Peak Viscosity	0.955	−0.156	0.182
X_10_	Trough Viscosity	0.928	−0.213	0.179
X_11_	Final Viscosity	0.939	0.046	0.251
X_12_	Breakdown	0.960	−0.133	0.183
X_13_	Setback	0.892	0.189	0.277
X_14_	Inorganic arsenic	0.941	−0.315	−0.029
X_15_	Cd	0.851	0.226	−0.1
X_16_	Total phenols	−0.963	0.223	−0.057
X_17_	Dietary fiber	−0.943	0.298	0.006
X_18_	VB_2_	−0.948	0.147	0.063
X_19_	VB_1_	−0.892	0.316	−0.179
X_20_	VE	−0.953	0.238	−0.03
Eigenvalue		14.79	2.30	1.25
Variance contribution rate (%)		73.93	11.49	6.25
Cumulative variance contribution rate (%)		73.93	85.42	91.67

After rotating the load matrix of the principal components, the load coefficient approaches 1 or 0, enabling the principal components to explain and name the variables better. As shown in Table 3, PC1 integrates most of the indicator information, including hardness, gumminess, chewiness, peak viscosity, trough viscosity, final viscosity, breakdown, inorganic arsenic, dietary fiber, VB_2_, and VE. Dietary fiber, total phenols, VB_2_ and VE had a negative distribution, indicating that the larger dietary fiber, total phenols, VB_2_ and VE, the smaller the values of the principal component. PC2 primarily integrates two types of information: springiness and pasting temperature. Pasting temperature had a positive distribution, whereas springiness had a negative distribution, reflecting the cooking quality of rice. PC3 integrated the information on adhesiveness, cohesiveness, and resilience. Adhesiveness had a negative distribution, while cohesiveness and resilience had a positive distribution. According to the component factor‐loading matrix in Table 3, the functions of each principal component are as follows:

$$\begin{array}{c} F_{1}=0.24{\text{9X}}_{1}+0.22{\text{4X}}_{2}-0.00{\text{6X}}_{3}-0.18X_{4}+0.24{\text{8X}}_{5}\\ \;+0.24{\text{5X}}_{6}-0.15{\text{2X}}_{7}+0.08{\text{3X}}_{8}+0.24{\text{8X}}_{9}+0.24{\text{1X}}_{10}\\ \;+0.24{\text{4X}}_{11}+0.24{\text{9X}}_{12}+0.23{\text{2X}}_{13}+0.24{\text{4X}}_{14}\\ \;+0.22{\text{1X}}_{15}-0.25X_{16}-0.24{\text{5X}}_{17}-0.24{\text{6X}}_{18}-0.23{\text{2X}}_{19}-0.24{\text{8X}}_{20} \end{array}$$

$$\begin{array}{c} F_{2}=0.11{\text{0X}}_{1}-0.03{\text{1X}}_{2}-0.44{\text{6X}}_{3}-0.40{\text{5X}}_{4}+0.14{\text{3X}}_{5}\\ \;+0.18{\text{4X}}_{6}-0.27{\text{2X}}_{7}+0.48{\text{3X}}_{8}-0.10{\text{3X}}_{9}-0.14X_{10}\\ \;+0.03X_{11}-0.08{\text{8X}}_{12}+0.12{\text{4X}}_{13}-0.20{\text{7X}}_{14}+0.14{\text{9X}}_{15}\\ \;+0.14{\text{7X}}_{16}+0.19{\text{6X}}_{17}+0.09{\text{7X}}_{18}+0.20{\text{8X}}_{19}+0.15{\text{7X}}_{20} \end{array}$$

$$\begin{array}{c} F_{3}=-0.10{\text{5X}}_{1}-0.37X_{2}-0.36{\text{6X}}_{3}+0.28{\text{4X}}_{4}-0.09{\text{6X}}_{5}\\ \;-0.07{\text{9X}}_{6}+0.50{\text{1X}}_{7}+0.37{\text{1X}}_{8}+0.16{\text{3X}}_{9}+0.16X_{10}\\ \;+0.22{\text{4X}}_{11}+0.16{\text{3X}}_{12}+0.24{\text{7X}}_{13}-0.02{\text{6X}}_{14}-0.08{\text{9X}}_{15}\\ \;-0.05{\text{1X}}_{16}+0.00{\text{5X}}_{17}+0.05{\text{6X}}_{18}-0.16X_{19}-0.02{\text{7X}}_{20} \end{array}$$

A comprehensive quality score function was constructed for the 11 samples by dividing the eigenvalues of principal components 1, 2, and 3 by the sum of their eigenvalues as weights. Its functional expression is

$$Z=0.81F_{1}+0.13F_{2}+0.07F_{3}$$

According to the formula, the comprehensive scores of rice with different DOM was −6.34–3.48. J‐9 had the highest score of 3.48 and J‐0 had the lowest score of −6.34. Table S1 showed the scoring of 11 rice with different degree of milling.

#### 3.5.3 Cluster Analysis

CA groups research subjects with similar feature information in the same category without removing their original information; all categories are equally important (Singh et al. 2013). Rice samples with different DOM and indicators were classified to better demonstrate the correlation between the different samples and indicators using CA (Figure 4B).

Rice was divided into three groups. Group one consisted of J‐0 rice, which is brown rice. The dietary fiber and total phenols of brown rice were high because of the complete layer. However, it has poor hardness, viscosity, and gelatinization temperature and is characterized by high hardness, gelatinization temperature, and low viscosity. Group 2 comprised J‐1, J‐2, J‐3, and J‐4. Although they were grounded, the degree of grinding was relatively light, and a certain amount of the cortex was retained. Group 3 is J‐5, J‐6, J‐7, J‐8, J‐9, J‐10. The layer was mostly removed, and most of the nutritional indicators were lost. However, the edible quality has greatly improved. Overall, this classification model is reasonable.

The rice quality indicators were divided into three groups. As three principal components (PCs) with eigenvalues > 1 were extracted from PCA, it was reasonable to categorize all assessment indicators into three groups (Zheng et al. 2022). The 1st group of indicators included final viscosity, setback, trough viscosity, breakdown, and peak viscosity, which indicate the pasting properties of rice. The 2nd group of indicators included pasting temperature and springiness, which reflect the cooking quality. The 3rd group included hardness, gumminess, chewiness, adhesiveness, total phenols, VE, dietary fibers, Cd, inorganic arsenic, VB_2_, VB_1_, resilience, and cohesiveness, which reflect the texture and nutritional quality of rice. This is consistent with the PCA results.

## 4 Conclusion

The whole‐grain diet has been increasingly advocated in recent years to regulate lifestyle habits and reduce risk factors for certain diseases and inflammation. In this study, as the DOM increases, the bran of rice is gradually milled, the endosperm is exposed, and some bioactive substances such as vitamins, dietary fiber, and total phenols are gradually transferred to the rice bran. However, heavy metal elements show a decreasing trend, and the gelatinization and texture characteristics after cooking show a positive trend. This indicates that although milling removes the bioactive components of brown rice, it is beneficial for heavy metal elements and cooking characteristics. However, how to balance the nutritional loss caused by rice milling and the improvement of cooking characteristics is addressed by developing a rice quality evaluation system for different DOMs using CRA, PCA, and CA. The results revealed correlations between the rice quality indicators. To address these overlaps, we performed PCA to simplify the indicators and selected three principal components that accounted for a cumulative contribution rate of 91.67%, which clarified the variation in rice quality. The CA results also grouped the rice and quality indicators into three clusters, which are consistent with the PCA findings. Finally, J‐9 (DOM 9%) scored the highest in the built model. Thus, the evaluation model established in this study provides a theoretical foundation for the development of a quality evaluation system for the major japonica rice varieties in northern China.

## Author Contributions

Shan Zhang: writing‐original draft, writing – review and editing, visualization, funding acquisition. Bin Hong: visualization, methodology. Di Yuan: visualization, conceptualization. Shan Shan: software, methodology. Jingyi Zhang: data curation. Shiwei Gao: software. Qing Liu: investigation. Dapeng Chen: resources. Weiwei Yin: supervision. Chuanying Ren: writing – review and editing, project administration, funding acquisition.

## Conflicts of Interest

The authors declare no conflicts of interest.

## Supporting information


**Figure S1:** Microstructure of rice grains in different DOM, (a)–(k) are the surfaces of rice grain samples with DOM of 0%, 1%, 2%, 3%, 4%, 5%, 6%, 7%, 8%, 9%, and 10%, respectively.
**Table S1:** The comprehensive score of the rice with different degree of milling.

## Data Availability

The data that support the findings of this study are available on request from the corresponding author. The data are not publicly available due to privacy or ethical restrictions.
