# Supplementary material for: Establishment of a Comprehensive Quality Evaluation Model for Japonica Rice With Different Degrees of Milling
Source: Food Sci Nutr. 2025 Sep 4;13(9):e70908. doi: 10.1002/fsn3.70908 (PMC12411562; doi:10.1002/fsn3.70908)
Supplement: Supplementary file 1 — Figure S1: Microstructure of rice grains in different DOM, (a)–(k) are the surfaces of rice grain samples with DOM of 0%, 1%, 2%, 3%, 4%, 5%, 6%, 7%, 8%, 9%, and 10%, respectively. Table S1: The comprehensive score of the rice with different degree of milling. [file FSN3-13-e70908-s001.docx]

**Supplementary material**

**Establishment of a comprehensive quality evaluation model for japonica rice with different degrees of milling**

Shan Zhang ^a, b, c^, Bin Hong ^a, b, c^, Di Yuan ^a, b, c^, Shan Shan ^a, b, c^, Jingyi Zhang ^a, b, c^, Shiwei Gao^d^, Qing Liu^d^, Dapeng Chen^e^, Weiwei Yin^e^, Chuanying Ren ^a, b, c, *^

^a^ Food Processing Research Institute, Heilongjiang Academy of Agricultural Sciences, Harbin 150086, China;

^b^ Heilongjiang Province Key Laboratory of Food Processing, Harbin 150086, China;

^c^ Heilongjiang Province Engineering Research Center of Whole Grain Nutritious Food, Harbin 150086, China

^d^ Suihua Branch of Heilongjiang Academy Agricultural Sciences, Suihua, 152000, China;

^e^ Heilongjiang Vocational College of Agricultural Technology, Jiamusi 154007, China

Corresponding authors at: Food Processing Research Institute, Heilongjiang Academy of Agricultural Sciences, Harbin 150086, China

Tel: +86-0451-86610253.

E-mail address: rcxuan@foxmail.com (C. Y. Ren).


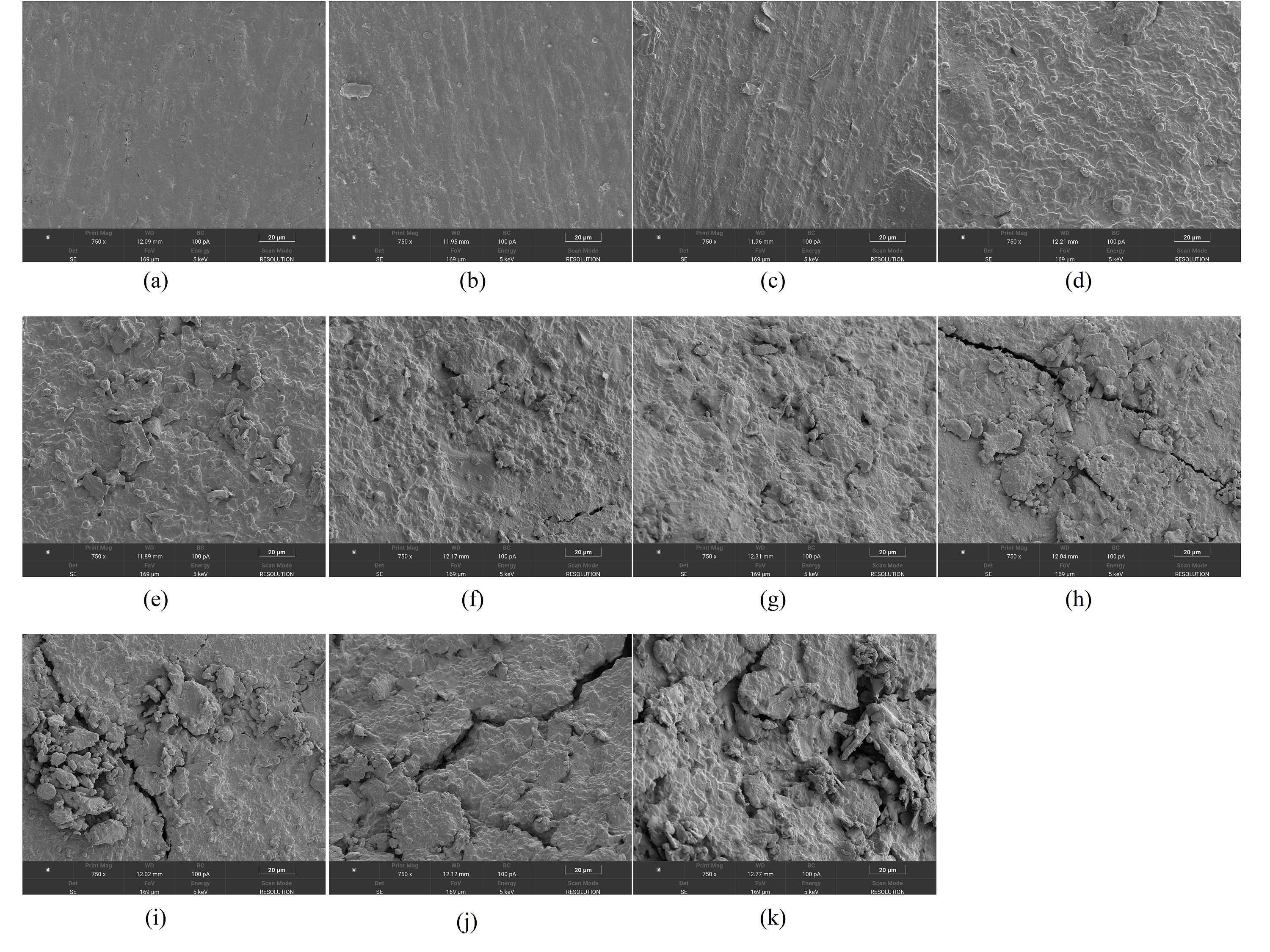


Fig. S1. Microstructure of rice grains in different DOM, (a) - (k) are the surfaces of rice grain samples with DOM of 0%, 1%, 2%, 3%, 4%, 5%, 6%, 7%, 8%, 9%, and 10%, respectively

**Table S1** The comprehensive score of the rice with different degree of milling

| DOM | F_1_ | F_2_ | F_3_ | Comprehensive score | Rank |
| --- | --- | --- | --- | --- | --- |
| J-0 | -7.42 | -2.23 | -0.57 | -6.34 | 11 |
| J-1 | -4.98 | 0.18 | -0.62 | -4.05 | 10 |
| J-2 | -2.92 | 0.44 | 1.72 | -2.18 | 9 |
| J-3 | -1.79 | 1.60 | -0.65 | -1.29 | 8 |
| J-4 | 0.37 | 2.58 | 0.55 | 0.67 | 6 |
| J-5 | 0.78 | 0.26 | -0.21 | 0.65 | 7 |
| J-6 | 1.56 | 0.50 | 0.46 | 1.36 | 5 |
| J-7 | 2.80 | 1.00 | -0.24 | 2.38 | 4 |
| J-8 | 3.52 | -1.01 | -0.70 | 2.67 | 2 |
| J-9 | 4.61 | -0.99 | -1.78 | 3.48 | 1 |
| J-10 | 3.48 | -2.32 | 2.04 | 2.66 | 3 |

F_1_ to F_3_ represent the scores of the 1st to 3rd principal component.
